# Evaluating the DMFT and dmft indices in people with epilepsy: A systematic review and meta-analysis

**DOI:** 10.1097/MD.0000000000043611

**Published:** 2025-08-08

**Authors:** Sobhan Agheshteh Sabzevar, Amir Valaei-Barhagh, Seyed Amirali Ghasemi, Nafise Agheshteh Sabzevar, Soheil Hassanipour

**Affiliations:** a Student Research Committee, School of Dentistry, Guilan University of Medical Sciences, Rasht, Iran; b School of Dentistry, Islamic Azad University of Isfahan (Khorasgan) Branch, Isfahan, Iran; c Gastrointestinal and Liver Diseases Research Center, Guilan University of Medical Sciences, Rasht, Iran.

**Keywords:** DMF index, dmft, DMFT, epilepsy, meta-analysis, oral health, systematic review

## Abstract

**Background:**

Epilepsy is a chronic neurological disorder characterized by recurrent seizures, which can significantly affect various aspects of health, including oral health. This systematic review and meta-analysis aimed to evaluate the oral health status of individuals with epilepsy by analyzing the decayed, missing, and filled teeth (DMFT) and dmft indices across multiple studies.

**Methods:**

A comprehensive literature search was conducted across several databases, including PubMed, Scopus, Google Scholar, Web of Science, and ProQuest, resulting in the inclusion of 7 studies that met predefined criteria. The studies included case-control, cohort, and cross-sectional designs published until October 30, 2024. Data extraction focused on DMFT and dmft scores among participants with epilepsy compared to control groups. The analysis employed standardized mean differences to assess the impact of epilepsy on dental health.

**Results:**

The analysis revealed a statistically significant increase in the DMFT index for permanent teeth among epileptic patients compared to controls (SMD = 0.403; 95% CI: 0.057 to 0.749; *P* = .022), indicating a higher prevalence of dental caries in this population. Conversely, the dmft index for primary teeth did not show a statistically significant difference (SMD = 0.132; 95% CI: −0.22 to 0.484; *P* = .463). Factors contributing to these findings include the effects of antiepileptic medications, seizure frequency, and challenges in maintaining oral hygiene.

**Conclusion:**

This study highlights the concerning disparity in oral health among individuals with epilepsy, particularly regarding permanent teeth. The findings underscore the necessity for targeted dental care interventions and preventive strategies tailored to meet the unique needs of this population. By improving awareness and access to dental care, healthcare providers can enhance the overall quality of life for individuals living with epilepsy. Further research is warranted to explore effective interventions that can mitigate dental health risks in this vulnerable group.

## 1. Introduction

Oral health issues represent one of the most prevalent public health concerns.^[1]^ Despite interventions implemented in recent years, oral health remains concerningly inadequate, and studies have shown elevated prevalence rates of dental caries among children and adolescents under 18 years old.^[2]^ Enhancements in oral health can be achieved through behavioral changes, improving socioeconomic status, increasing education levels among individuals and parents, and enhancing access to health insurance services. Special attention should be given to implementing dental caries prevention programs targeting individuals with specific systemic conditions, due to their poor behavioral habits and lower educational levels.^[3]^ The WHO’s global oral health reports have a clear conclusion indicating that the global oral health status is alarming and requires immediate action.^[4]^ Dental caries represent a dynamic biological process leading to the irreversible destruction of hard dental tissues due to acids produced from bacterial glycolysis of dietary carbohydrates.^[5]^

For over 70 years, the decayed-missing-filled teeth index (DMFT for permanent teeth and dmft for primary teeth) has been recognized globally as a significant metric for assessing oral health in various communities.^[6,7]^

Epilepsy is a chronic neurological disorder characterized by a persistent tendency to experience seizures.^[8]^ Seizures resulting from epilepsy are transient events caused by excessive or abnormal neural activity in the brain. A diagnosis of epilepsy is made when an individual has experienced seizures that are not provoked by identifiable triggers, indicating a pathological and sustained predisposition to recurrent seizures. Specifically, epilepsy is diagnosed when the individual meets the following criteria: at least 2 unprovoked seizures occurring more than 24 hours apart, one unprovoked (or reflex) seizure, and a probability of further seizures similar to the general recurrence risk (at least 60%) after 2 unprovoked seizures, occurring over the next 10 years; and presence of an epilepsy syndrome.^[9]^ Suppose clinical features, EEG (electroencephalogram) findings, or imaging results increase the likelihood of further seizures to 60%. In that case, individuals are classified as having epilepsy, with their clinical risk of recurrence statistically comparable to those already diagnosed with the condition.^[10]^ Epilepsy is the 4th most common neurological disorder, affecting approximately one in 10 individuals at some point in their lives. In the United States, one in every 26 people is diagnosed with epilepsy, contributing to a global prevalence of around 65 million individuals living with this condition, with an annual increase of approximately 2.4 million new cases.^[11,12]^ Seizures impact about 10% of the global population and lead to epilepsy in 1% to 2% of individuals worldwide.^[13]^ Epilepsy affects both genders and individuals across all age groups, exhibiting a global prevalence pattern. The incidence and prevalence rates are slightly higher in males compared to females and peak among older adults due to increased rates of strokes, neurological diseases, and tumors within this demographic.^[8]^ The overall prognosis for epilepsy is favorable for most patients; however, not all types require treatment.^[10]^ Medication adherence is crucial for achieving optimal therapeutic outcomes and effectively managing seizures in patients with epilepsy (PWE).^[8,14]^

Researches indicate that PWE may face additional risks for developing dental caries due to various factors, including antiepileptic medications, the effects of seizures on motor skills, and oral hygiene practices.^[15]^ Moreover, if these patients do not receive regular dental follow-ups, they tend to exhibit higher rates of decayed and missing teeth compared to the general population.^[16]^ Liquid oral medication serves as an additional source of sugar for children with chronic illnesses requiring long-term care; these children are at increased risk for dental caries due to this factor.^[15]^ Additionally, studies have indicated that patients suffering from epilepsy exhibit notably poorer gum health compared to their healthy counterparts.^[17,18]^

Understanding the oral health challenges faced by individuals with epilepsy is crucial for developing effective preventive strategies and treatment protocols. This systematic review will not only fill existing gaps in the literature but also highlight the need for targeted dental care interventions for epileptic patients. By establishing a clearer picture of their oral health status through DMFT and dmft evaluations, healthcare providers can better address the unique needs of this vulnerable population, ultimately improving their overall quality of life.

## 2. Materials and methods

### 2.1. Ethics declaration

Ethical approval does not apply to this systematic review and meta-analysis article, as it is based on previously conducted studies.

### 2.2. Design

The results were reported using the PRISMA statement methodology to enhance the transparency and completeness of systematic reviews and meta-analyses in healthcare research.^[19]^ The protocol for this study was published in the International Prospective Register of Systematic Reviews, or PROSPERO. The registration PROSPERO ID was CRD42025648793.

### 2.3. Search strategy

The literature search was conducted across 5 different online databases, including Web of Science/ISI, Scopus, PubMed, ProQuest, and the first 10 pages of Google Scholar using relevant MeSH headings and keywords related to “DMFT” and “epilepsy.” Two researchers (SH and SA) independently performed the searches, and any discrepancies were resolved through discussion. Additionally, we reviewed all references to ensure a thorough search. EndNote software version 20 was utilized to manage the search process effectively. The search strategy used in the PubMed, Web of Science/ISI, Scopus, ProQuest, and Google Scholar databases is provided in the Supplementary Material (Supplemental Digital Content, https://links.lww.com/MD/P559).

### 2.4. Inclusion and exclusion criteria

Inclusion criteria were as follows:

1) Participants with epilepsy
2) Participants’ DMFT or dmft has been evaluated.
3) Case-control, cohort, and cross-sectional studies.
4) Studies carried out up to October 30, 2024.
5) Studies written in English

To ensure the review was focused, the eligibility criteria were designed to address a specific question using the PICOT framework (population, intervention, comparison, outcome). The PICOT components were defined as follows:

- P (Population): Participants with epilepsy
- I (Intervention): Not applicable in this study
- C (Comparison): Healthy participants
- O (Outcome): DMFT or dmft

### 2.5. Exclusion criteria

Exclusion criteria included studies involving patients with other systemic disorders or conditions that could impact oral health (such as other neurological disorders), non-English language publications, review articles, papers lacking full text, conference abstracts, studies assessed to have a high risk of bias, and studies that did not provide adequate data on the DMFT or dmft index.

### 2.6. Data extraction

Three independent researchers (AV, AG, and NAS) selected relevant studies based on established inclusion criteria. After refining the search strategy and eliminating duplicate publications, they carefully screened the titles, abstracts, and full texts to identify and exclude irrelevant studies. Data from all eligible articles were extracted using a predefined checklist. This checklist encompassed the first author’s name, year of publication, journal name, country of the study, type of study, number of participants, sample size, mean age, gender distribution, and quality assessment.

### 2.7. Quality assessment

The quality of the articles was evaluated using a checklist created by The Joanna Briggs Institute (JBI).^[20]^ This checklist is designed to assess the methodological quality of studies and evaluate how effectively they have addressed potential biases in their design, implementation, and analysis. Each paper was thoroughly examined for its relevance to the data and its methodological rigor.

Data extraction and quality assessment were performed by the 3 independent researchers (AV, SAS, and SH).

### 2.8. GRADE assessment

The current meta-analysis was evaluated using the grade (grading of recommendations, assessment, development, and evaluation) framework. This assessment considered factors such as study design, risk of bias, precision, consistency, directness, and publication bias, categorizing the quality of evidence as “high,” “moderate,” “low,” or “very low.”

### 2.9. Statistical analysis

Heterogeneity among the studies and their combination was evaluated using the Cochran test and the I² statistic, respectively. In cases where heterogeneity was detected, a random effects model utilizing the inverse variance method was applied; conversely, a fixed effects model was used when no heterogeneity was present. The standardized mean difference was employed to compare the DMFT or dmft scores of participants with epilepsy against those without epilepsy. A power analysis was conducted for each outcome, and all analyses were performed using CMA version 3 statistical software.

## 3. Results

### 3.1. Study selection

**Figure 1.**
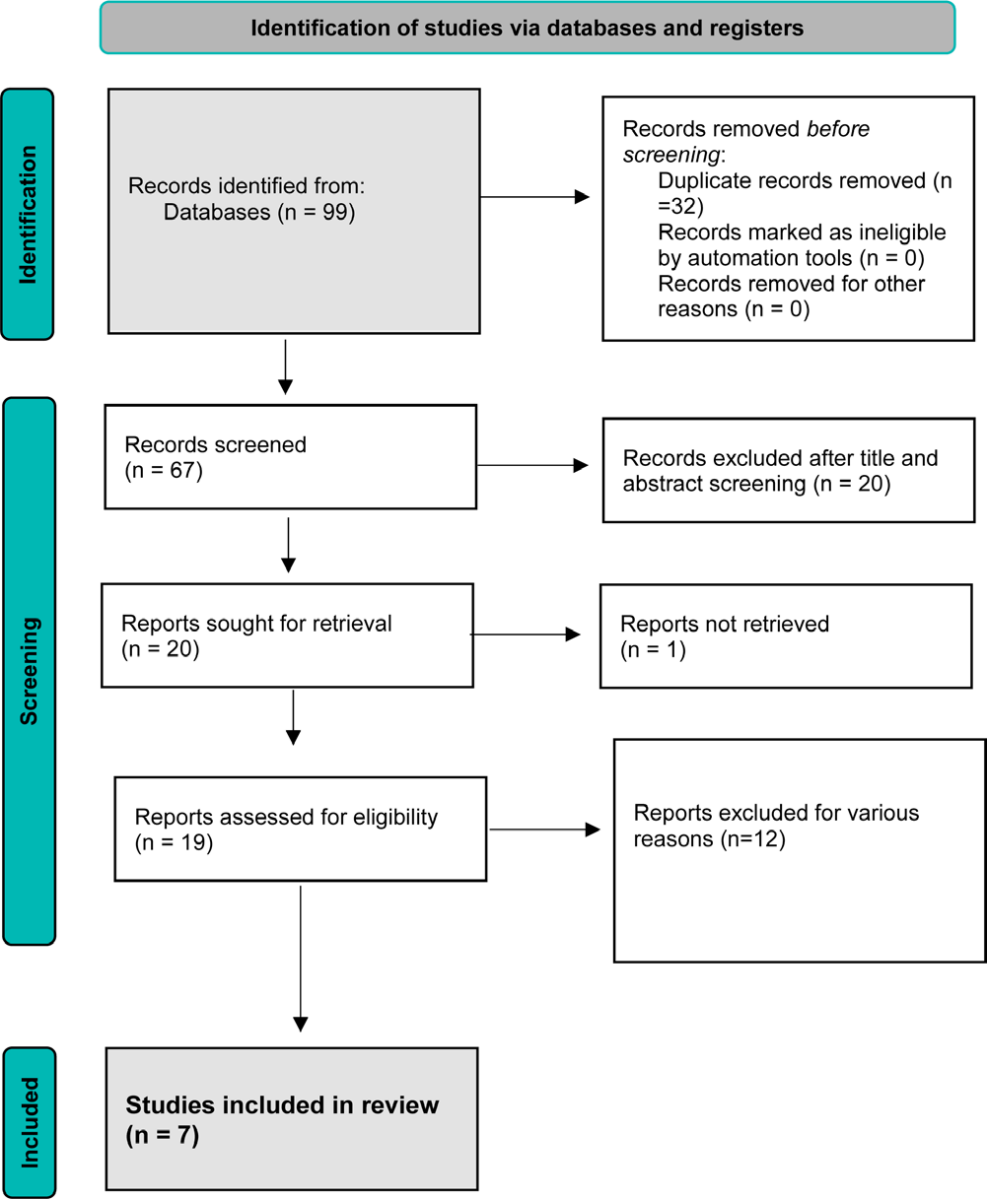
Flow diagram of search methodology and literature selection process.

Figure 1 illustrates the comprehensive study selection process. Initially, a total of 99 articles were identified. After removing duplicates, 67 articles remained for further assessment. During the screening of titles and abstracts, 20 articles were found to be relevant. However, one article, “Prevalence of Dental Caries in Handicapped Children of Calcutta” could not be accessed in full text. Consequently, 19 articles were selected for a thorough evaluation based on their full texts. Reviewing these full texts determined that several studies lacked the necessary information for our analysis. As a result, only 7 articles met all inclusion criteria and were included in our final study.

### 3.2. Quality assessment

**Figure 2.**
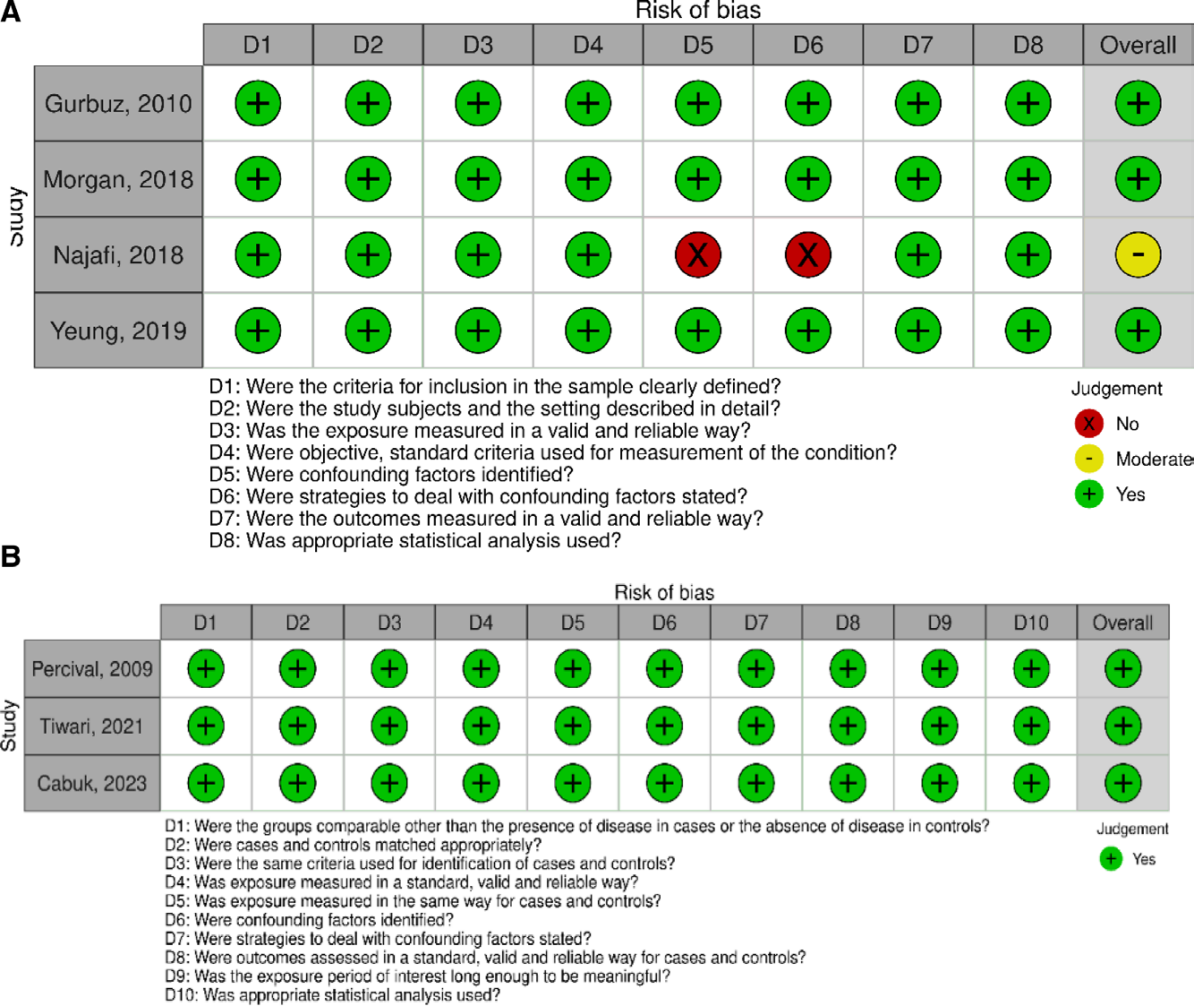
The results of the risk of bias assessment. (A) cross-sectional studies, (B) case–control studies.

Figure 2 provides detailed information on the quality assessment of the included studies. According to the JBI checklist, 6 studies were classified as high quality and one as moderate quality.

### 3.3. Characteristics of the studies

The key characteristics of the included studies are summarized in Table 1. Among these, 2 studies were conducted in Turkey, one each in Iran, England, Egypt, and China. The overall mean sample size was 167, with individual studies ranging from 70 to 442 participants.

**Table 1:** Main characteristics of included studies.

First author, year of publication	Country	Journal	Type of study	Sample size	Age category	DMFT*/dmft†	Quality of study
Percival, 2009^[36]^	England	European Archives of Paediatric Dentistry	Case-control	78	4–16	DMFT/dmft	10/10
Gurbuz, 2010^[37]^	Turkey	Pediatrics International	Cross-sectional	442	4–15	DMFT/dmft	8/8
Morgan, 2018^[38]^	Egypt	International Journal of Pediatric Dentistry	Cross-sectional	180	6–12	DMFT/dmft	8/8
Najafi, 2018^[34]^	Iran	Journal of Craniomaxillofacial Research	Cross-sectional	120	12–69	DMFT	6/8
Yeung, 2019^[18]^	China	Journal of investigative and clinical dentistry	Cross-sectional	70	3–18	DMFT/dmft	8/8
Tiwari, 2021^[26]^	India	International Journal of Clinical Pediatric Dentistry	Case-control	200	5–16	DMFT/dmft	10/10
Cabuk, 2023^[25]^	Turkey	Journal of Cukurova Anesthesia and Surgical Sciences	Case-control	82	18–66	DMFT	10/10

* Decayed, missing, and filled teeth (index for permanent teeth).

† Decayed, missing, and filled teeth (index for primary teeth).

### 3.4. GRADE assessment

According to the grade assessment of the current meta-analysis, this study has a “high” quality of evidence. For additional information, please refer to the grade table in the Supplementary Material (Supplemental Digital Content, https://links.lww.com/MD/P559).

## 4. Outcomes

### 4.1. DMFT

**Figure 3.**
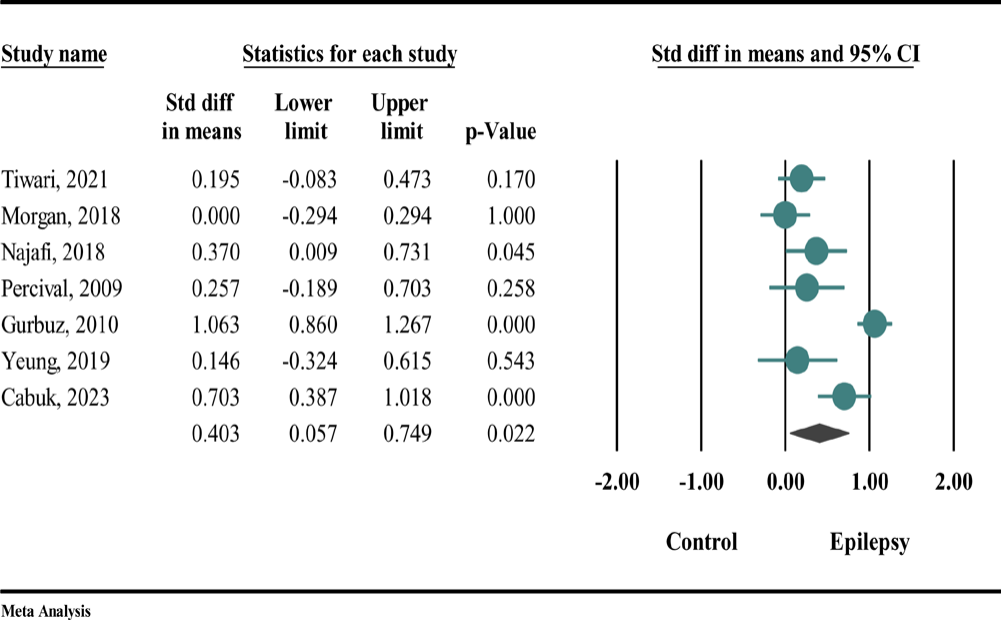
The difference in DMFT between participants with epilepsy and those without epilepsy. DMFT = decayed, missing, and filled teeth (index for permanent teeth).

As shown in Figure 3, epilepsy led to an increase in DMFT, though the association was statistically significant (SMD = 0.403; 95% CI: 0.057 to 0.749; *P* = .022). Due to the I^2^ = 88.072, the random effects model was utilized.

### 4.2. dmft

**Figure 4.**
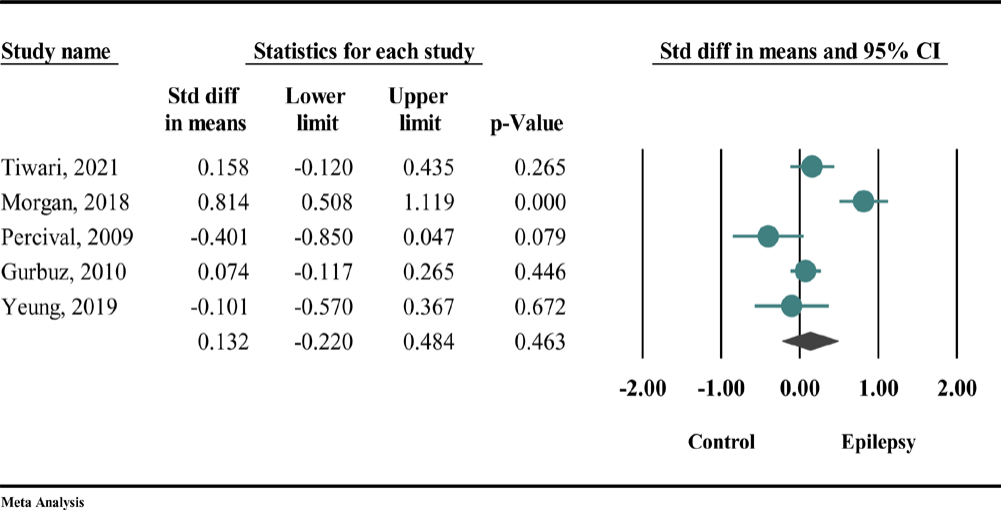
The difference in dmft between participants with epilepsy and those without epilepsy. Dmft = decayed, missing, and filled teeth (index for primary teeth).

Compared to the control group, epilepsy increased dmft levels, though the effect was not statistically significant (SMD = 0.132; 95% CI: −0.22 to 0.484; *P* = .463) (Fig. 4). Due to the I^2^ = 84.329, the random effects model was utilized.

### 4.3. Sensitivity analysis

**Figure 5.**
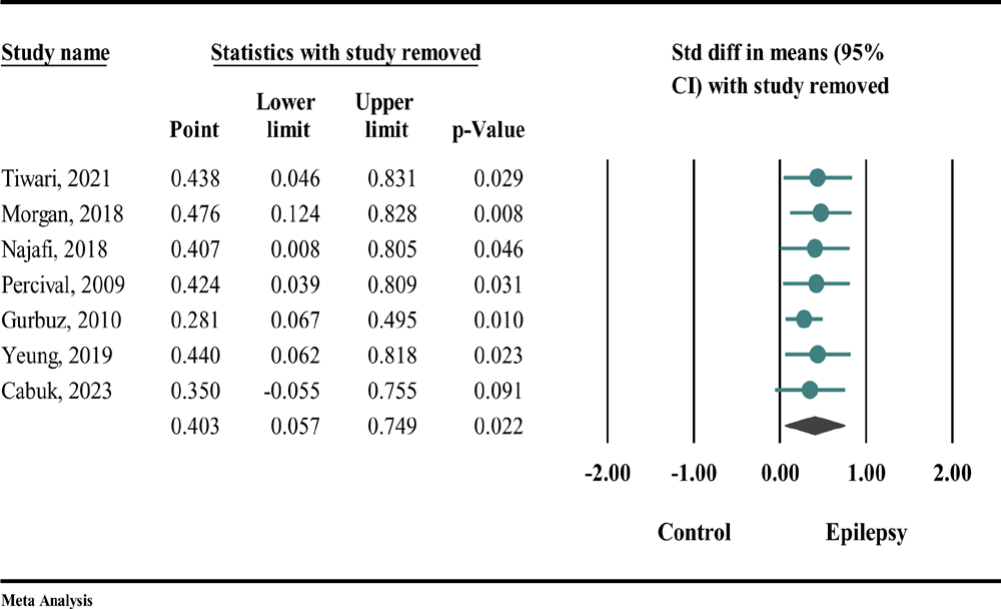
Sensitivity analysis employing “one removal study” among the studies in which they utilized the DMFT index. DMFT = decayed, missing, and filled teeth (index for permanent teeth).

Given the variability observed in the results, we conducted a sensitivity analysis utilizing the “one study removal” method to pinpoint potential sources of heterogeneity. The findings from the sensitivity analysis in Figure 5 indicated that the association remained stable, with no significant changes noted.

**Figure 6.**
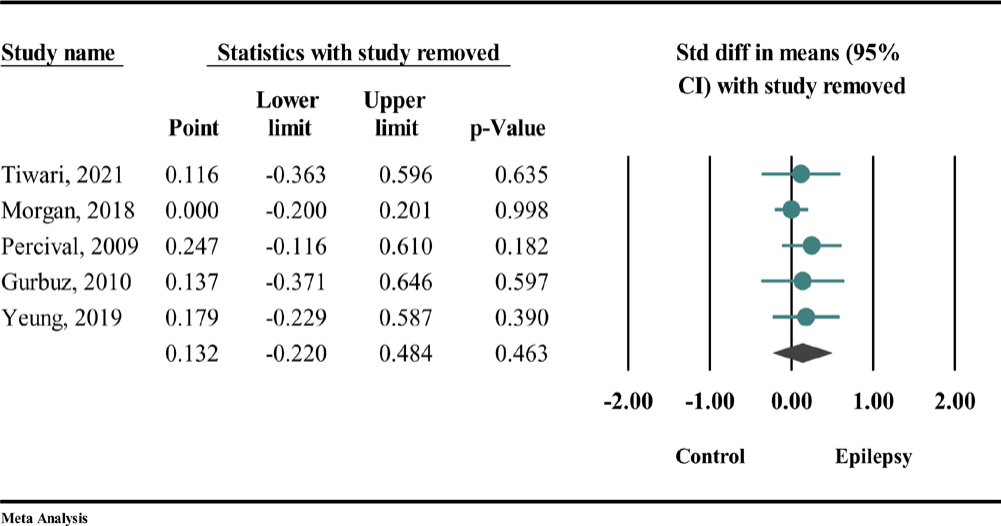
Sensitivity analysis employing “one removal study” among the studies in which they utilized the dmft index. dmft = decayed, missing, and filled teeth (index for primary teeth).

The results of the sensitivity analysis presented in Figure 6 demonstrate that the association remained stable, with no significant changes detected, except for the study conducted by Morgan.

### 4.4. Subgroup analysis

**Figure 7.**
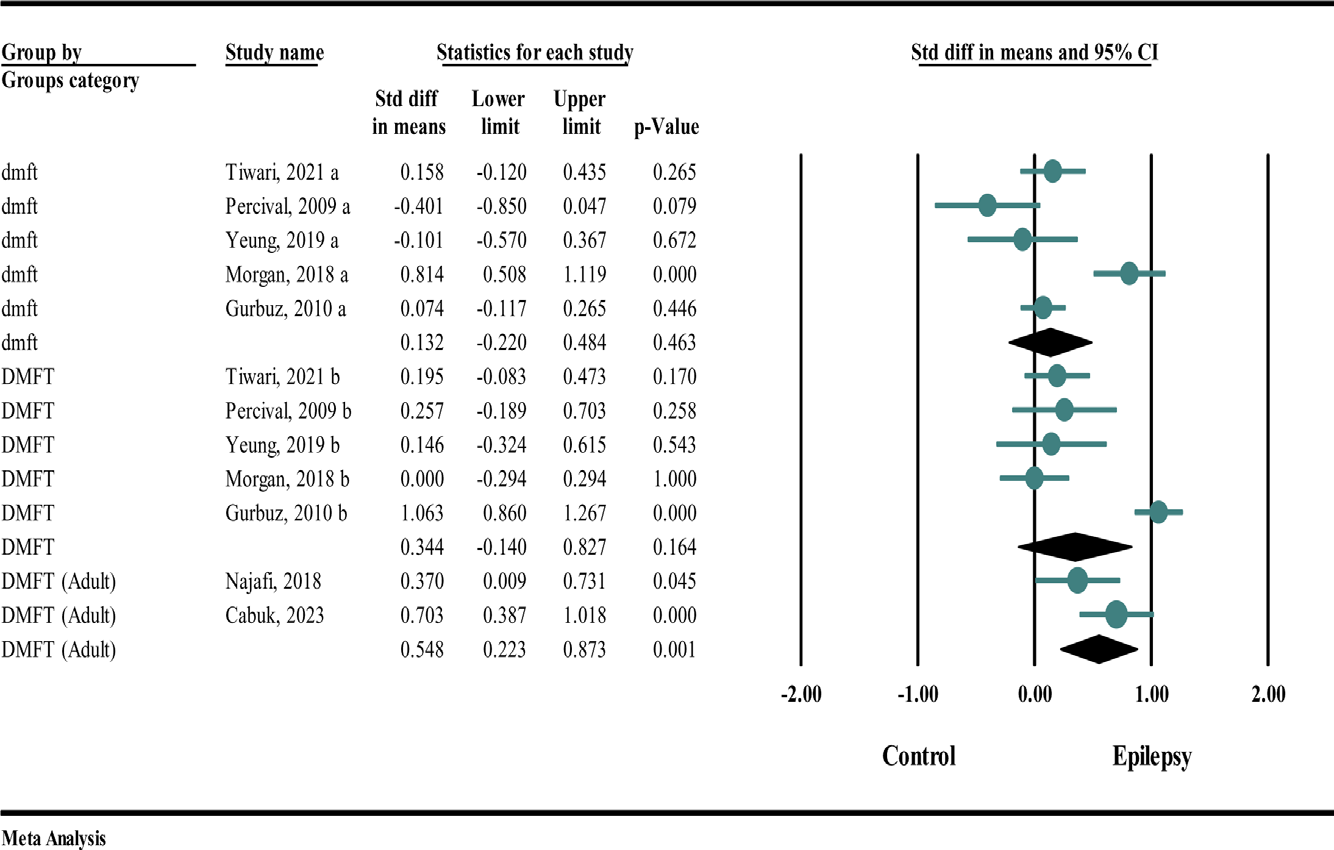
Forest plot of subgroup analysis.

Obtained results from subgroup analysis revealed that the DMFT in adult participants significantly increases (SMD 0.548; 95% CI: −0.223 to 0.873; *P* < .001). Also, an increase in dmft or DMFT in children or adolescents is observed due to epilepsy, but it is not statistically significant (SMD dmft 0.132; 95% CI: −0.220 to 0.484; *P* = .463) (SMD DMFT 0.334; 95% CI: −0.140 to 0.827; *P* = .164) (Fig. 7).

### 4.5. Power analysis

**Figure 8.**
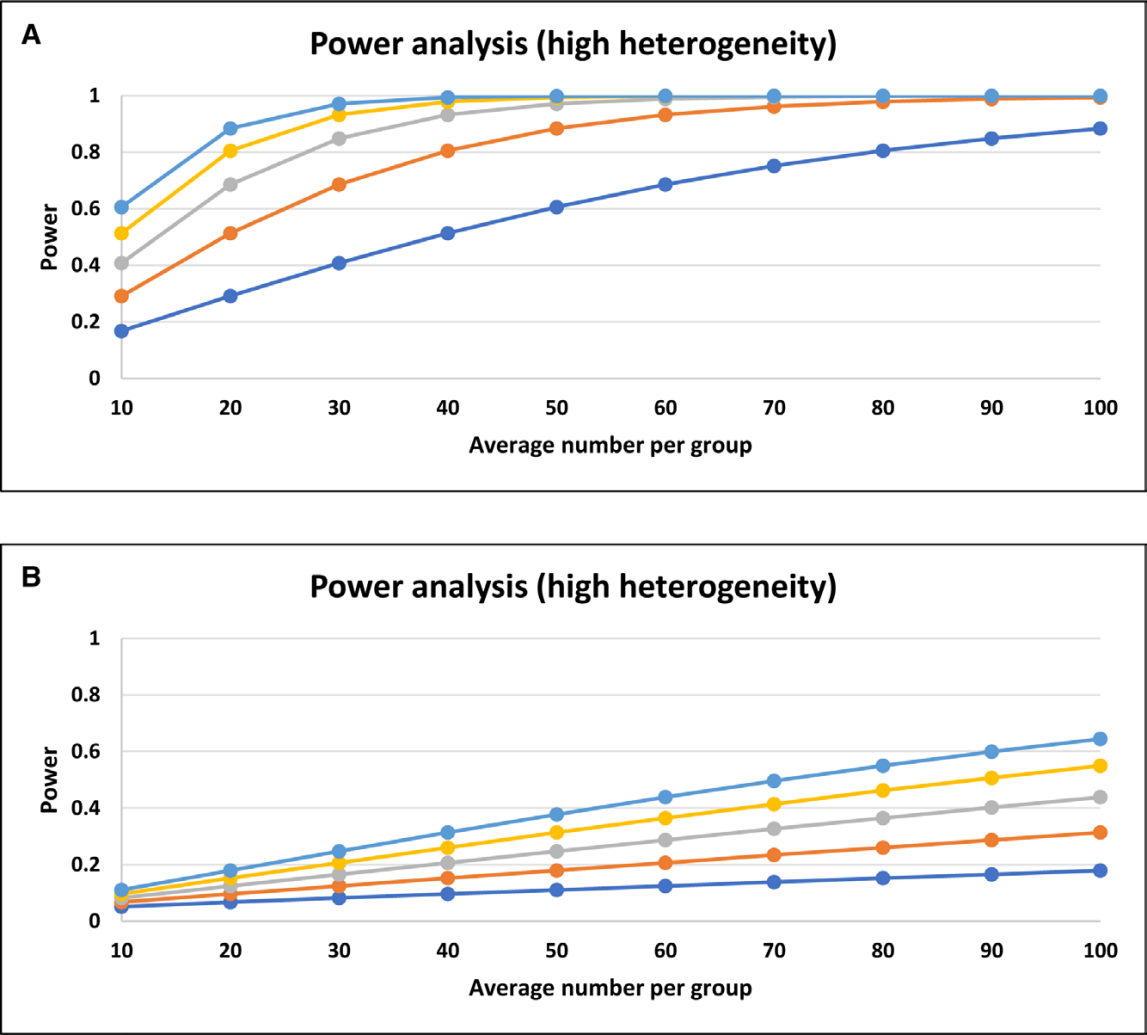
Association between epilepsy and DMFT or dmft and power analysis (A; DMFT, B; dmft). DMFT = decayed, missing, and filled teeth (index for permanent teeth), dmft = decayed, missing, and filled teeth (index for primary teeth).

A power analysis was performed using Hedges’ g statistic (DMFT = 0.943, dmft = 0.175) (Fig. 8A and B).

## 5. Discussion

Individuals with special healthcare needs frequently overlook their dental hygiene, resulting in a decline in oral health.^[21]^ Patients with well-managed seizures can typically receive dental care on an outpatient basis, but dentists must be well-informed about epilepsy and the medications used to treat it. A comprehensive medical history is vital, particularly for PWE, who may also have other health issues, and consultations with neurologists are recommended for tailored care.^[22]^ Understanding the interplay between systemic health issues and oral health can guide dentists in tailoring their preventive measures and treatment plans more effectively. As epilepsy can complicate dental care due to various factors, including medication side effects and potential neglect of oral hygiene, healthcare providers must remain vigilant in monitoring these patients.

This review seeks to clarify the extent to which epilepsy affects dental health, particularly caries prevalence as measured by the DMFT index.

The DMFT index is a crucial measure for assessing dental health, specifically indicating the count of decayed, missing, and filled teeth. This index was developed by Klein and Palmer in 1938.^[23]^ However, it has limitations, as it only assesses cavitated caries that have reached the dentin and does not account for the progression of caries or treatment requirements. Early-stage incipient caries lesions should not be overlooked, as they can often be reversed with preventive treatments when identified promptly.^[24]^

In the pioneering study conducted by Çabuk et al in Turkey, involving 164 participants (82 epileptic patients and 82 controls), significant differences were noted in oral health outcomes. The epilepsy group exhibited a mean of 7.27 ± 3.994 decayed teeth compared to 4.04 ± 2.202 in the control group (*P* < .001). Additionally, the number of missing teeth was significantly higher in the epilepsy group (5.71 ± 5.891) versus controls (3.01 ± 3.763), with a notable overall DMFT score of 15.05 ± 7.128 in epileptic patients compared to 10.55 ± 5.589 in controls (*P* < .001).^[25]^ Research indicates that individuals with epilepsy frequently experience higher rates of dental issues, including cavities, periodontal disease, and gingival hyperplasia, often linked to antiepileptic drugs such as phenytoin.^[25]^ One study highlighted that epilepsy patients were almost 5 times more likely to have poor oral hygiene and 4 times more prone to periodontitis compared to those without epilepsy.^[26,27]^ Other studies corroborate these findings, indicating that individuals with epilepsy experience elevated rates of dental issues such as cavities and periodontal disease, often exacerbated by antiepileptic medications like phenytoin.^[28]^

Moreover, many patients report anxiety related to dental visits, particularly if they have experienced seizures in a dental setting before. This anxiety can lead to avoidance of dental care altogether, creating a vicious cycle of poor oral health and increased anxiety about treatment.^[29]^

According to the findings from Tiwari et al, no significant difference was observed between DMFT and dmft in the studied population. This outcome regarding the index related to permanent teeth contrasts with the results of this study, which may be attributed to the fact that the population under investigation consisted of children aged 5 to 16 years. It appears that epilepsy and the effects of antiepileptic medication, along with poor oral hygiene, require a longer duration to manifest an impact following the eruption of permanent teeth.^[26]^

The most striking observation from our analysis is the higher DMFT index in permanent teeth of epileptic patients compared to primary teeth. This disparity suggests that the impact of epilepsy on dental health may be more pronounced in permanent dentition. Interestingly, the differences in DMFT index for primary teeth were not statistically significant, indicating that epilepsy might have a less severe effect on deciduous dentition.

To understand the underlying reasons for this discrepancy, it’s crucial to examine the structural differences between primary and permanent teeth. Research has shown that primary teeth have thinner enamel and dentin layers compared to permanent teeth.^[30]^ This structural variation may contribute to the different DMFT index patterns observed in our study. Primary teeth also have a larger pulp chamber relative to their size, which can lead to faster progression of dental caries.^[31]^ However, permanent teeth, being exposed to the oral environment for a longer duration, have thicker enamel and dentin layers that initially provide better protection against caries. However, prolonged exposure to risk factors associated with conditions like epilepsy may lead to a higher DMFT index over time, as these factors can accumulate and affect dental health significantly.^[32]^

Contrary to popular belief, seizures resulting from epilepsy have minimal significance regarding the physical trauma inflicted on oral and dental tissues, and they do not independently affect the DMFT index. Consequently, studies indicate that a combination of factors associated with this condition can ultimately influence the oral health status of patients.^[26,33]^

### 5.1. Factors influencing DMFT in epileptic patients

Several factors may contribute to the higher DMFT index in permanent teeth of epileptic patients:

1. Medication effects: Long-term use of antiepileptic drugs (AEDs) can lead to xerostomia (dry mouth), reducing the protective effects of saliva and increasing caries risk. Research has shown that xerostomia is a common side effect of AEDs, significantly contributing to dental caries in PWE.^[33]^
2. Seizure frequency: Frequent seizures may impact oral hygiene practices and increase the risk of dental trauma. Studies indicate that seizures can lead to oro-facial injuries, including dental trauma, as patients may not be able to protect their mouths during an episode.^[26]^
3. Dietary habits: Some epileptic patients may have dietary restrictions or preferences that affect their oral health. Dietary habits can be influenced by medication side effects and seizure management strategies, which may lead to increased sugar intake or neglect of oral hygiene.^[34]^
4. Oral hygiene challenges: Motor impairments or cognitive difficulties associated with epilepsy might hinder effective oral hygiene practices. The challenges faced by individuals with epilepsy in maintaining oral hygiene are well-documented, highlighting the need for tailored dental care strategies.^[35]^

### 5.2. Strengths and limitations

This systematic review and meta-analysis have several limitations that should be acknowledged. Firstly, the number of studies included in the analysis was limited to 7, which may restrict the generalizability of the findings. The variability in study designs, sample sizes, and populations across the included studies could introduce bias and affect the overall results. For instance, while some studies focused on children, others included adults, potentially influencing the DMFT and dmft indices due to developmental differences in dental health. Secondly, the quality of the studies varied, with one study classified as moderate quality while the others were deemed high quality according to the JBI checklist. This variation in methodological rigor may impact the reliability of the conclusions drawn from this review. Additionally, some studies lacked comprehensive data on confounding variables such as socioeconomic status, dietary habits, and adherence to dental care practices, which could further influence oral health outcomes. Finally, the cross-sectional nature of many included studies limits causal inferences regarding the impact of epilepsy on oral health. Longitudinal studies are needed to better understand how epilepsy and its associated factors influence dental health over time.

Despite these limitations, this systematic review and meta-analysis possess several strengths that enhance its contribution to the existing literature. The comprehensive search strategy employed across multiple databases ensured a thorough identification of relevant studies, minimizing the risk of overlooking important research. Furthermore, adherence to PRISMA guidelines for systematic reviews enhances the transparency and rigor of the methodology. The inclusion of diverse populations from various countries allows for a broader understanding of how epilepsy affects oral health across different cultural and healthcare contexts. This diversity strengthens the external validity of the findings and highlights potential variations in dental health outcomes among epileptic patients globally. Additionally, by focusing specifically on DMFT and dmft indices, this review provides a clear quantitative assessment of dental caries prevalence among individuals with epilepsy.

## 6. Conclusion

This systematic review and meta-analysis indicate no statistically significant differences in the dmft index for primary teeth between people with epilepsy and control groups; however, the significantly higher DMFT scores for permanent teeth among those with epilepsy warrant attention. This suggests that the effects of epilepsy on dental health may be more pronounced in permanent dentition. These results highlight the urgent need for greater awareness among dental professionals regarding the specific oral health challenges encountered by PWE. Future research should further investigate these relationships and develop effective strategies to improve oral health within this at-risk population.

## Acknowledgments

We extend our gratitude to all the authors of the studies included in this systematic review and meta-analysis. Their valuable contributions have significantly contributed to the findings and conclusions of this research.

## Author contributions

**Conceptualization:** Soheil Hassanipour, Sobhan Agheshteh Sabzevar.

**Data curation:** Amir Valaei-Barhagh, Soheil Hassanipour, Sobhan Agheshteh Sabzevar.

**Investigation:** Sobhan Agheshteh Sabzevar, Seyed Amirali Ghasemi, Nafise Agheshteh Sabzevar.

**Methodology:** Amir Valaei-Barhagh, Seyed Amirali Ghasemi, Soheil Hassanipour.

**Software:** Soheil Hassanipour.

**Supervision:** Soheil Hassanipour.

**Writing – original draft:** Sobhan Agheshteh Sabzevar, Amir Valaei-Barhagh, Nafise Agheshteh Sabzevar.

## Supplementary Material


