# Supplementary material for: Evaluating the DMFT and dmft indices in people with epilepsy: A systematic review and meta-analysis
Source: Medicine (Baltimore). 2025 Aug 8;104(32):e43611. doi: 10.1097/MD.0000000000043611 (PMC12338231; doi:10.1097/MD.0000000000043611)
Supplement: Supplementary file 1 [file medi-104-e43611-s001.docx]

**Supplementary material**

**Search strategy:**

**PubMed: (16 articles)**

(("epilepsy"[Title/Abstract] OR "seizure*"[Title/Abstract]) OR (("Seizures"[Mesh]) OR "Epilepsy"[Mesh])) AND ("DMFT*"[Title/Abstract] OR "dmft*"[Title/Abstract])

**ISI (Web Of Science): (10 articles)**

(ALL=("Epilepsies" OR "Seizure Disorder*" OR "Cryptogenic" OR "Epilepsy" OR "Cryptogenic Epilepsy" OR "Cryptogenic Epilepsies" OR "Aura*" OR "Awakening Epilepsy" OR "Seizure" OR "Jacksonian Seizure" OR "Single Seizure*" OR "Atonic Absence Seizure*" OR "Focal Seizure*" OR "Partial Seizure*" OR "Generalized Seizure*" OR "Sensory Seizure*" OR "Auditory Seizure*" OR "Convulsive Seizure*" OR "Motor Seizure*" OR "Gustatory Seizure*" OR "Olfactory Seizure*" OR "Somatosensory Seizure*" OR "Vertiginous Seizure*" OR "Vestibular Seizure*" OR "Visual Seizure*" OR "Non-Epileptic Convulsion*" OR "Nonepileptic Seizure*" OR "Non-Epileptic Seizure*" OR "Non Epileptic Seizure*" OR "Nonepileptic Seizure*" OR "Complex Partial Seizure*" OR "Epileptic Seizure*" OR "Generalized Absence Seizure*" OR "Tonic-Clonic Seizure*" OR "Generalized Tonic-Clonic Seizure*" OR "Clonic Seizure*" OR "Clonic Seizure*" OR "Tonic Seizure*")) AND ALL=((“DMFT*” OR “dmft*”))

**ProQuest: (2 articles)**

summary("Epilepsies" OR "Seizure Disorder*" OR "Cryptogenic" OR "Epilepsy" OR "Cryptogenic Epilepsy" OR "Cryptogenic Epilepsies" OR "Aura*" OR "Awakening Epilepsy" OR "Seizure" OR "Jacksonian Seizure" OR "Single Seizure*" OR "Atonic Absence Seizure*" OR "Focal Seizure*" OR "Partial Seizure*" OR "Generalized Seizure*" OR "Sensory Seizure*" OR "Auditory Seizure*" OR "Convulsive Seizure*" OR "Motor Seizure*" OR "Gustatory Seizure*" OR "Olfactory Seizure*" OR "Somatosensory Seizure*" OR "Vertiginous Seizure*" OR "Vestibular Seizure*" OR "Visual Seizure*" OR "Non-Epileptic Convulsion*" OR "Nonepileptic Seizure*" OR "Non-Epileptic Seizure*" OR "Non Epileptic Seizure*" OR "Nonepileptic Seizure*" OR "Complex Partial Seizure*" OR "Epileptic Seizure*" OR "Generalized Absence Seizure*" OR "Tonic-Clonic Seizure*" OR "Generalized Tonic-Clonic Seizure*" OR "Clonic Seizure*" OR "Clonic Seizure*" OR "Tonic Seizure*") AND summary(“DMFT*” OR “dmft*”)

**Scopus: (28 articles)**

TITLE-ABS-KEY ( "Epilepsies" OR "Seizure Disorder*" OR "Cryptogenic" OR "Epilepsy" OR "Cryptogenic Epilepsy" OR "Cryptogenic Epilepsies" OR "Aura*" OR "Awakening Epilepsy" OR "Seizure" OR "Jacksonian Seizure" OR "Single Seizure*" OR "Atonic Absence Seizure*" OR "Focal Seizure*" OR "Partial Seizure*" OR "Generalized Seizure*" OR "Sensory Seizure*" OR "Auditory Seizure*" OR "Convulsive Seizure*" OR "Motor Seizure*" OR "Gustatory Seizure*" OR "Olfactory Seizure*" OR "Somatosensory Seizure*" OR "Vertiginous Seizure*" OR "Vestibular Seizure*" OR "Visual Seizure*" OR "Non-Epileptic Convulsion*" OR "Nonepileptic Seizure*" OR "Non-Epileptic Seizure*" OR "Non Epileptic Seizure*" OR "Nonepileptic Seizure*" OR "Complex Partial Seizure*" OR "Epileptic Seizure*" OR "Generalized Absence Seizure*" OR "Tonic-Clonic Seizure*" OR "Generalized Tonic-Clonic Seizure*" OR "Clonic Seizure*" OR "Clonic Seizure*" OR "Tonic Seizure*" ) AND TITLE-ABS-KEY ( "DMFT*" OR "dmft*" )

**Google Scholar: (43 articles)**

“epilepsy” AND (“dmft” OR “DMFT”)
